# Analgesic Efficacy of Ketoprofen Transdermal Patch versus Ibuprofen Oral Tablet on Postendodontic Pain in Patients with Irreversible Pulpitis: A Randomized Clinical Trial

**DOI:** 10.1155/2023/8549655

**Published:** 2023-06-07

**Authors:** Saeede Zadsirjan, Amirhossein Toghrolian, Nazanin Zargar

**Affiliations:** ^1^Department of Endodontics, School of Dentistry, Shahid Beheshti University of Medical Sciences Tehran, Tehran, Iran; ^2^Student Research Committee, Guilan University of Medical Sciences, Guilan, Iran

## Abstract

**Materials and Methods:**

In this randomized clinical trial, 64 patients who had mandibular first and second molars with irreversible pulpitis were randomly divided into two groups (*n* = 32) by stratified permuted block randomization. The experimental group used 60 mg KTP every 6 hours, and the control group received 400 mg ibuprofen tablets every 6 hours for 1 day. The severity of pain experienced by patients was quantified before and at 2, 4, 8, 12, 24, and 48 hours after endodontic treatment, using the numerical rating scale (NRS). Data were analyzed by using the *t*-test, Mann–Whitney test, and generalized estimating equation (GEE) (alpha = 0.05).

**Results:**

The pain score was not significantly different between the two groups at the baseline or any other postoperative time point (*P* > 0.05). The reduction in the pain score was significant in both groups from 2 to 10 hours and 10 to 48 hours, postoperatively (*P* < 0.001). The interaction effect of time and group was not significant on the postoperative pain score in the abovementioned time intervals, and the pattern of pain reduction was the same over time in both groups (*P* > 0.05).

**Conclusion:**

Both KTP and ibuprofen effectively decreased postendodontic pain. Considering the comparable pattern of pain reduction, KTP can be used as an alternative to ibuprofen tablets for effective pain control after endodontic treatment of mandibular first and second molars with irreversible pulpitis.

## 1. Introduction

Pain is an unpleasant sensory experience, which is often associated with possible or actual tissue injury [1]. Prevention and control of pain is an important aspect in endodontic treatments. The prevalence of postendodontic pain is relatively high [2], and it ranges from 3% to 58% [3]. A successful dental treatment requires the appropriate use of professional techniques and control of postoperative pain [4].

Postoperative administration of analgesics is often imperative to decrease postendodontic pain especially in teeth with irreversible pulpitis [5]. Patients with higher levels of preoperative pain often experience higher levels of postendodontic pain, compared with asymptomatic patients [6]. Also, a strong correlation exists between the pulp status and the level of postoperative pain [7]. Gotler et al. [8] indicated that postendodontic pain was significantly higher in teeth with vital pulp that underwent root canal therapy compared with teeth with necrotic pulp.

A high level of postoperative pain is a common concern for both patients and dental clinicians, and it can underline patients' trust in treatment. Thus, analgesics, particularly nonsteroidal anti-inflammatory drugs (NSAIDs), are commonly prescribed before and after endodontic treatment [9, 10]. NSAIDs exert their anti-inflammatory and analgesic effects by inhibiting the cyclooxygenase enzyme and synthesis of prostaglandins [11].

Ibuprofen is among the most commonly prescribed NSAIDs for arthritis, menstrual pain, postoperative pain, edema, and fever. Approximately 80% of ibuprofen is absorbed through the gastrointestinal system when taken orally, and the analgesia onset occurs 30 minutes after use. It is metabolized in the liver and has a half-life of 1.8 to 2 hours. Also, it is mainly excreted in urine and slightly through the bile [12].

Ketoprofen is another NSAID that has a similar structure to ibuprofen since it has a P-phenylpropionic group [12]. Like Ibuprofen, it is metabolized in the liver and has a half-life of 2 to 2.5 hours. Since prostaglandins stimulate the pain receptors, inhibition of their synthesis by ketoprofen leads to analgesia [13].

Analgesics can be used through oral, injection, inhalation, and transdermal routes. Oral medication intake is associated with possible hepatic primary metabolism, resulting in subsequent elimination of a large part of medication prior to its systemic absorption [14]. Moreover, oral medication intake results in high plasma levels of drug and the associated risks of gastrointestinal complications, renal failure, hepatotoxicity, sodium retention, hypertension, and developing resistance to antihypertensive drugs [15, 16].

Transdermal patch is a relatively novel form of medication delivery. The patch adheres to the skin and releases a certain dose of drug that passes through the skin and underlying tissues to reach the blood vessels [17]. The advantages of transdermal patches include nonhepatic primary metabolism, lower plasma concentration, and subsequently lower systemic cytotoxicity and side effects [18, 19]. Moreover, transdermal patches are better accepted by patients [20].

Considering the limited number of studies on the efficacy of analgesic transdermal patches for postendodontic pain control, this study aimed to compare the efficacy of ketoprofen transdermal patch (KTP) and ibuprofen tablets for pain control after endodontic treatment of mandibular first and second molars with irreversible pulpitis.

## 2. Materials and Methods

This study was conducted at the Shahid Beheshti University of Medical Sciences between February 2019 and April 2021. The study was approved by the Ethics Committee of this university (IR.SBMU.DRC.REC.1398.226) and registered in the Iranian Registry of Clinical Trials (IRCT20190716044230N1).

### 2.1. Trial Design

An intention-to-treat randomized clinical trial was designed in which the experimental group received KTP, while the control group received ibuprofen tablets to control postendodontic pain. The results were reported according to the guidelines of the Consolidate standards of Reporting Trials [21].

### 2.2. Participants, Eligibility Criteria, and Settings

The inclusion criteria were age between 18 and 65 years, ASA class I physical health status [22], having a mandibular first or second molar with irreversible pulpitis and moderate (pain score of 4 to 7) or severe (pain score of 8 to 10) pain according to the numerical rating scale (NRS), positive response to electric pulp test, moderate to severe abnormal response with/without a prolonged response to cold test (confirming the diagnosis of irreversible pulpitis for the respective tooth), and no history of gastrointestinal bleeding or problem, no allergy to aspirin-like drugs such as ibuprofen, and no analgesic intake within the past 48 hours prior to admission.

The exclusion criteria were emergency cases, presence of radiolucency on the radiograph, presence of edema and fistula, use of more than 2 anesthetic cartridges for inferior alveolar nerve block or supplemental injections during the endodontic procedure, noticing pulpal necrosis in general or in one canal after initiation of treatment, not being able to complete endodontic treatment of the respective tooth within a 2-hour single session, iatrogenic errors during treatment (such as perforation, accidental overinstrumentation, or extension of root filling material into the periapical region or beyond the working length), postoperative edema and the need for postoperative antibiotic therapy, and noncompliant patients not precisely reporting the postoperative pain scores.

The sample consisted of 64 eligible patients with mandibular first or second molars with irreversible pulpitis and moderate to severe pain presenting to the Endodontics Department of School of Dentistry, Shahid Beheshti University of Medical Sciences.

### 2.3. Interventions

Eligible patients were enrolled after signing informed consent forms. They were briefed about the study protocol and objectives and the advantages and possible side effects of medications.

Demographic information of patients was recorded, and their preoperative level of pain and anxiety was quantified using an NRS. Accordingly, they were requested to select a number from 0 to 10 that best described their pain level (0 indicated no pain at all, while 10 indicated most severe pain imaginable). A graded NRS with information below each number was used in this study for easy understanding and high accuracy [23]. Mandibular molars were evaluated by the electric pulp test (The Elements Diagnostic Unit; Sybron Endo, Glendora, CA, USA) and cold test (Roeko Endo-Frost; Roeko Langenau, Germany). Patients with positive response to the electric pulp test and moderate (scores 4–7) or severe (scores 8–10) pain scores were diagnosed with irreversible pulpitis and enrolled. Sensitivity to percussion was also recorded.

All procedures were entirely performed under rubber dam isolation. Patients received 1 cartridge of 2% lidocaine with 80,000 epinephrine (Persocaine, Darupakhsh, Tehran, Iran) for inferior alveolar nerve block. If the patients had pain during access cavity preparation or root canal therapy, they received a PDL supplemental injection of lidocaine. An apex locator (Root ZX, Morito Corporation, Kyoto, Japan) was used for determination of the working length. The working length was determined 1 mm shorter than the radiographic apex and confirmed by a periapical radiograph. In addition, 1 mL of 2.5% sodium hypochlorite was used for root canal irrigation after using each file to the working length. First, the coronal part of the canals was flared with ^#^2 and ^#^3 Gates-Glidden drills, and then SP1 rotary system (Shanghai Fanta Dental Materials Co., Ltd., China) was used by the crown-down technique to accomplish root canal preparation. The mesial canals were prepared to ^#^30 with 4% taper, and the distal canals were prepared to ^#^35 with 4% taper. The root canals were then dried with paper points (Aradent, Iran) and filled with gutta-percha and AH26 sealer (Dentsply DeTrey, Konstanz, Germany) by the lateral compaction technique. All patients underwent endodontic treatment by two calibrated postgraduate students of endodontics under the supervision of an experienced endodontist. The teeth were then temporarily restored with temporary restorative material (Cavit; 3M, USA). Immediately after the treatment, the patients were instructed to record their level of pain at 2, 4, 8, 12, 24, and 48 hours after treatment using the NRS. The patients then randomly received an envelope containing pain medication and instructions for use. To ensure that the patients had fully understood the instructions for use of medications after opening the envelopes, the instructions for use were once again explained to patients and the patients used their first dose right after completion of their treatment [24]. The patients were requested to use 1 KTP (in KTP group) or 1 ibuprofen tablet (in ibuprofen tablet group) every 6 hours for the first 24 hours, post-treatment. The patients were instructed to apply the KTP on a hairless area such as the forearm. The patients also received an envelope containing 10 acetaminophen tablets (500 mg) [25] as a rescue drug and asked to contact the researcher and use the rescue drug if the KTP or ibuprofen tablets did not alleviate their pain. Such patients were excluded from the study. Also, to ensure that the patients fill out the NRS form regularly, the researcher contacted each patient every 6 hours and reminded them to record their pain level (as mentioned earlier, at 2, 4, 8, 12, 24, and 48 hours after treatment). The forms were then collected from patients, and the pain severity was classified as no pain (score 0), mild pain (scores 1–3), moderate pain (scores 4–6), or severe pain (scores 7–10).

Side effects and complications reported by patients were also recorded.

### 2.4. Outcomes (Primary and Secondary)

The main objective of this study was to compare the efficacy of KTP and ibuprofen tablets for pain control after endodontic treatment of mandibular first and second molars with irreversible pulpitis. The effects of sensitivity to percussion preoperatively, type of tooth, and gender of patients on postoperative pain were also assessed as the secondary outcome measures.

### 2.5. Sample Size Calculation

The sample size was calculated to be 28 in each group (a total of 56) according to a previous study by Murthykumar and Varghese [26] assuming alpha = 0.05, beta = 0.2, and study power of 80%. To increase the accuracy and control for the possible dropouts, 64 patients were enrolled.

### 2.6. Interim Analyses and Stopping Guidelines

No interim analyses were performed, and no stopping guidelines were established.

### 2.7. Randomization

The patients were randomly divided into two groups by stratified permuted block randomization with block size = 4 using sequentially numbered, sealed, opaque envelopes [27]. Since 64 patients were enrolled, four groups (*n* = 16) were considered for randomization: (I) males with a mandibular first molar with irreversible pulpitis, (II) males with a mandibular second molar with irreversible pulpitis, (III) females with a mandibular first molar with irreversible pulpitis, and (IV) females with a mandibular second molar with irreversible pulpitis. In each of the four groups, each patient randomly received KTP or ibuprofen tablets. Thus, first, four sequences of 16 of the two drugs were randomly created by filliping a coin. Next, 64 envelopes with aluminum covers (to mask the contents) were created and coded. The medications were placed in the envelopes and sealed. A copy of the list of blocks was created by an assistant (who had no involvement in the next phases of the study).

### 2.8. Blinding

A dental assistant randomly assigned the medication envelopes to patients. The dental clinician, researcher, and statistician who analyzed the data were all blinded to the group allocation of patients. Only the dental assistant who randomly assigned the coded envelopes to patients was aware of the contents of the envelopes. To ensure allocation concealment, sequentially numbered sealed opaque envelopes were used [27].

### 2.9. Statistical Analysis

Normal distribution of pain data was evaluated by the Kolmogorov–Smirnov test. The results showed that distribution of pain data was normal at 2, 4, 8, and 12 hours. Thus, comparisons at these time points were performed by the *t*-test. Pain data had non-normal distribution at 24 and 48 hours. Thus, comparisons at these time points were carried out by the Mann–Whitney test. The independent *t*-test was used to compare the level of pain of patients in the two groups before treatment. Spearman's correlation coefficient was applied to analyze the correlation of pain and anxiety before treatment with pain after treatment.

Considering the differences in distribution of data at different time points, the quantitative trend of pain over time was analyzed by generalized estimating equation (GEE). Since the trend of pain reduction over time was not linear, the Spline technique was used to analyze the trend of pain score change over time such that one line was considered from 2 to 10 hours and a second line was considered from 10 to 48 hours post-treatment. The overall form of the linear regression formula was as follows:

Mean pain score:

(1) $$\begin{array}{c} \beta_{0}+\beta_{1}drug+\beta_{2}time+\beta_{3}\left(time-10\right)+\beta_{4}drug*time+\beta_{5}drug*\left(time-10\right)\text{.} \end{array}$$

Finally, Fisher's exact test was used to compare the two groups regarding complete analgesia (NRS score 0) at different time points. All statistical analyses were carried out using SPSS version 20 at the 0.05 level of significance.

## 3. Results

**Figure 1:**
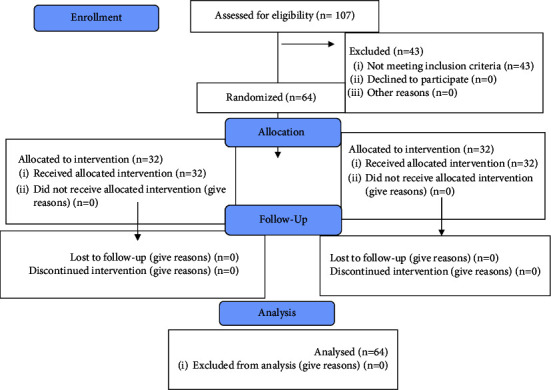
CONSORT flow diagram of the study.

The sample consisted of 64 patients including 32 males and 32 females, in two groups with equal gender distribution (16 males and 16 females in each group). The mean age of patients was 34.87 ± 11.16 years in the experimental group and 35.81 ± 11.19 years in the control group. The two groups had no significant difference regarding the mean age (*P* > 0.05).  Figure 1 shows the CONSORT flow diagram of patient selection and allocation.

### 3.1. Harms

No patients were harmed during the study.

### 3.2. Group Analyses

Table 1 presents the demographic variables in the two groups.  Table 2 indicates the mean pain score at different time points in the two groups. Considering the normal distribution of pain data at 2, 4, 8, and 12 hours, the two groups were compared regarding the pain score at these time points by the *t*-test, which revealed no significant difference at any time point (*P* > 0.05). At 24 and 48 hours, the groups were compared by the Mann–Whitney test, which revealed no significant difference (*P* > 0.05). Also, the Mann–Whitney test showed no significant difference in the pain score between the first and second molars (*P* > 0.05).

Spearman's correlation test showed a significant positive correlation between the anxiety score of patients before treatment with their preoperative (rho = 0.387, *P*=0.002) and postoperative pain at 2 (rho = 0.435, *P* < 0.001), 4 (rho = 0.371, *P*=0.003), 8 (rho = 0.354, *P*=0.004), 12 (rho = 0.352, *P*=0.004), and 24 (rho = 0.274, *P*=0.028) hours but not at 48 hours (rho = 0.181, *P*=0.152). Spearman's test also showed that irrespective of the group, the preoperative pain score of patients was significantly correlated with their postoperative pain score at 2 (rho = 0.401, *P*=0.001), 4 (rho = 0.560, *P* < 0.001), 8 (rho = 0.424, *P* < 0.001), 12 (rho = 0.462, *P* < 0.001), and 24 (rho = 0.392, *P*=0.001) hours but not at 48 hours (rho = 0.162, *P*=0.200).

Table 3 presents the correlation of preoperative pain and postoperative pain separately in each group. As shown, in the KTP group, preoperative pain had a significant positive correlation with postoperative pain up to 24 hours (*P* < 0.05). However, in the ibuprofen tablet group, the preoperative pain score had a significant positive correlation only with the pain score at 4 hours (*P*=0.027).

Comparison of patients with pain score 0 between the two groups at each time point by Fisher's exact test revealed no significant difference (*P* > 0.05).

Regarding sensitivity to percussion before treatment, 21 patients were evaluated (since the results of the percussion test had been recorded for only 21 patients); out of which, 5 were sensitive and 16 were not sensitive to percussion. The *t*-test showed that the difference in the pain score between sensitive patients and not sensitive to percussion was only significant at 2 hours postoperatively, and the pain score was higher in the group not sensitive to percussion (*P*=0.027). At other time points, pain was higher in the group not sensitive to percussion but not significantly (*P* > 0.05).

Two out of 32 patients in the ibuprofen tablet group reported gastrointestinal problems in the form of mild pain. Also, one patient in the KTP group experienced slight redness and itchiness when used the first KTP. Since he wanted to continue using the patch, he applied the next patch on another area with no problem.

**Figure 2:**
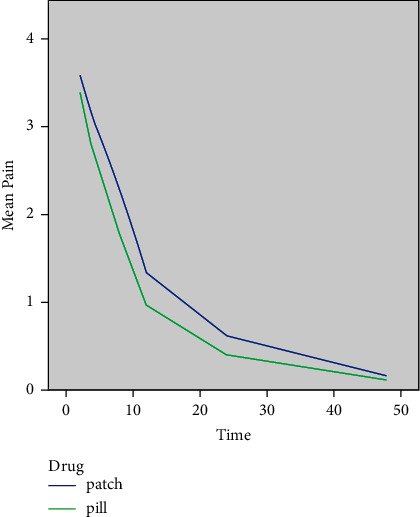
Mean pain score over time by the medication type.

Assessment of the pain score over time showed a nonlinear trend of reduction in pain over time in both groups (Figure 2). Thus, by using the Spline technique, two lines were considered: one from 2 to 10 hours and the other one from 10 to 48 hours.

Although the mean preoperative pain score was higher in the KTP group, the difference between the two groups was not significant (*P*=0.141). In both groups, the reduction in the pain score in both the first (2–10 hours) and second (10–48 hours) intervals was significant (*P* < 0.001 for both).

The interaction effect of time and medication was not significant at any interval in any group (*P*=0.432 for KTP and *P*=0.571 for tablet group), which means that the pattern of pain reduction over time was the same in both the groups.

**Figure 3:**
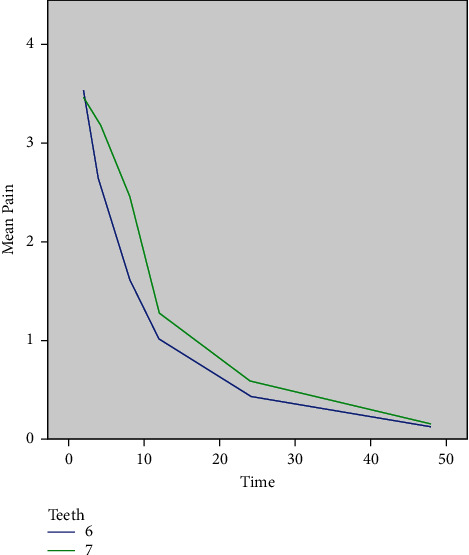
Mean pain score over time according to the molar type.

GEE was applied to assess the effect of type of tooth on postoperative pain and showed that although patients with a second molar with irreversible pulpitis had higher postoperative pain than those with a first molar with irreversible pulpitis (Figure 3), this difference was not significant (*P*=0.298).

**Figure 4:**
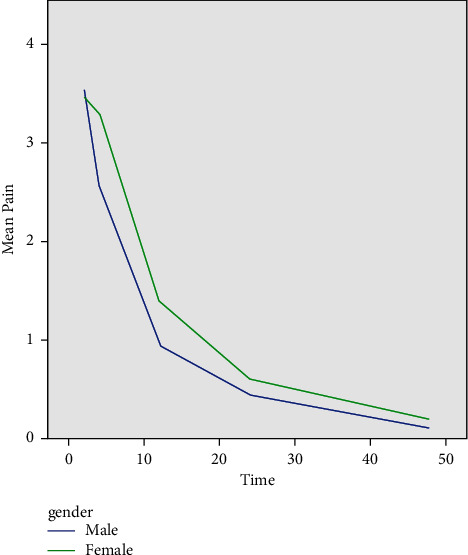
Mean pain score over time by gender.

GEE was also used to analyze the effect of gender, and it indicated that although females in both groups had a higher pain score than males (Figure 4), this difference did not reach statistical significance (*P*=0.472).

## 4. Discussion

This study compared the efficacy of KTP and ibuprofen tablets for pain control after endodontic treatment of mandibular first and second molars with irreversible pulpitis. The severity of preoperative pain and pulpal diagnosis are among the factors that can affect postoperative pain [10]. Thus, the two groups were standardized regarding these parameters. Also, regular administration of medications adopted in this study was due to the advantages of the regular technique such as lower pain experience by patients [28]. Selection of 400 mg ibuprofen tablets for the tablet group in this study was because 400 mg dosage of ibuprofen is required for its ceiling effect and that increasing the dosage does not significantly increase the analgesic efficacy [29]. Moreover, selection of 60 mg ketoprofen administered every 6 hours was according to a previous study [30].

In the present study, the NRS was used for quantification of pain due to its slightly superior efficacy to the visual analog scale [31]. Also, the NRS has higher reliability in both literate and illiterate patients [32]. It has been shown that the amount of apically extruded debris also affects the level of postoperative pain; thus, a rotary system with the crown-down technique was used in both groups in this study, which has been shown to minimize apical extrusion of debris [33]. The results revealed no significant difference in the pain score between the two groups at any time point, and both groups demonstrated a similar trend of pain reduction over time.

Evidence shows that preoperative pain and periapical allodynia (sensitivity to percussion) can significantly affect postendodontic pain [34]. In addition, the preoperative anxiety of patients increases their postendodontic pain experience [35]. Similarly, the present study showed significant positive correlations between preoperative pain and preoperative anxiety with postoperative pain up to 24 hours. However, patients sensitive to percussion experienced lower postoperative pain, and this correlation was significant at 2 hours post-treatment. The same results were reported by Parirokh et al.[38] who showed that preoperative pain had a greater effect than sensitivity to percussion on postendodontic pain. However, since the number of patients sensitive to percussion was low in the present study, further investigations are required on this topic.

Assessment of the effect of gender on the postoperative pain score by GEE in the present study showed that although females in both groups had a higher pain score than males, this difference did not reach statistical significance, which was in line with a previous study [37]. However, Gear et al. [38] used opioids for reduction of pain after oral surgical procedures and reported greater pain reduction in females than males. Variations in the results can be attributed to physiological differences of males and females and different individuals, as well as different pharmacodynamics of medications [39]. Also, pain perception is subjective and different individuals have different levels of pain perception thresholds, which can explain variations in the results.

GEE also assessed the effect of type of tooth on postoperative pain, and it showed that although patients with a second molar with irreversible pulpitis had higher postoperative pain than those with a first molar with irreversible pulpitis, this difference was not significant. No previous study is available on this topic to compare our results with.

In the present study, 2 patients in the ibuprofen tablet group had mild gastrointestinal pain, which was reported in 12 out of 16 patients in a study by Mangal et al. [40]. Difference between the present results and those of Mangal et al. [40] in this respect may be due to greater gastrointestinal problems caused by diclofenac compared with ibuprofen and inclusion of patients with no history of gastrointestinal problems and allergy to ibuprofen in the present study. In the study by Mangal et al. [40], no skin reaction to diclofenac patch was reported, while in the present study, one patient developed slight redness and itchiness at the site of KTP.

In the current study, although the mean preoperative pain score was higher in the KTP group, the difference between the two groups was not significant. In both groups, the reduction in the pain score in both the first (2–10 hours) and second (10–48 hours) intervals was significant. The interaction effect of time and medication was not significant at any interval in any group, which means that the pattern of pain reduction over time was the same in both the groups.

As mentioned earlier, several studies have evaluated the effects of NSAIDs in the form of transdermal patch on pain after tooth extraction [2, 14, 20, 41], periodontal surgery [26, 42], and maxillofacial surgical procedures [43, 44]. However, only one study was found on the efficacy of diclofenac sodium transdermal patch for postendodontic pain control [40], which reported results similar to the present findings and revealed that diclofenac patches had a comparable analgesic efficacy to ibuprofen tablets.

Moreover, controversy exists in the literature regarding the efficacy of transdermal patches. Three studies on pain control by oral intake of diclofenac sodium and its transdermal patch following periodontal flap surgery showed that its transdermal patch could have a higher [45], comparable [42], or lower [26] efficacy than its oral form. Such differences may be due to the use of different daily doses and flap elevation techniques. Also, studies on postextraction pain indicated that diclofenac patch can have equal [2, 41] or slightly lower efficacy in the first 24 hours and comparable efficacy at 48 hours [14] to diclofenac tablets. Although the severity and nature of periodontal and postextraction pains are different from postendodontic pain since a certain plasma concentration of analgesic is required for pain control (irrespective of type of pain), and transdermal patches have systemic effects as well, it may be concluded that such patches can be used to obtain plasma concentrations similar to oral tablets and successfully alleviate pain.

To the best of the authors' knowledge, this study is the first to assess the efficacy of KTP for postendodontic pain control, which is a major strength of this study. Assessment of the effect of gender on postoperative pain was another strength of this study.

This study had some limitations as well. Absence of a placebo group was a limitation of this study. However, it was not ethically possible to have a placebo group. Also, subjective nature of pain is another limitation, which cannot be controlled for. Moreover, the age range of the study population was high, which can affect the pain threshold.

Future studies on a larger sample size are required to increase the generalizability of the results.

## 5. Conclusion

Both KTP and ibuprofen effectively decreased postendodontic pain. Nonetheless, considering the optimal analgesic efficacy of KTP comparable to that of ibuprofen tablets and lower side effects of KTP, it may be used as an alternative to oral ibuprofen tablets for pain control following endodontic treatment of mandibular first and second molars with irreversible pulpitis.

## Figures and Tables

**Table 1:** Demographic variables in the two groups.

		Ibuprofen	Ketoprofen patch	Total
Gender	Males	16	16	32
	Females	16	16	32

Mandibular tooth	First molar	16	16	32
	Second molar	16	16	32

Age	Mean ± SD^*∗*^	34.875 ± 11.16	35.812 ± 11.19	35.343 ± 11.10

Anxiety score before treatment	Mean ± SD	3.81 ± 2.53	4.13 ± 2.44	3.97 ± 2.47

Pain score before treatment	Mean ± SD	5.845 ± 1.95	6.845 + 2.20	6.34 ± 2.12

^*∗*^SD: standard deviation.

**Table 2:** Mean pain score at different time points in the two groups.

Tooth number			Preoperative pain	2 hours	4 hours	8 hours	12 hours	24 hours	48 hours
First molar	KTP (*n* = 16)	Mean ± SD	6.63 ± 2.21	3.56 ± 3.07	2.81 ± 3.01	2 ± 2.50	1.25 ± 1.80	0.44 ± 0.89	0.06 ± 0.25
		Minimum	4	0	0	0	0	0	0
		Maximum	10	9	10	8	5	3	1
	Tablet (*n* = 16)	Mean ± SD	5.56 ± 1.75	3.5 ± 2.22	2.44 ± 1.96	1.25 ± 1.39	0.81 ± 1.37	0.44 ± 0.89	0.19 ± 0.54
		Minimum	4	1	0	0	0	0	0
		Maximum	8	8	6	5	5	3	2
		*P* value	0.143^*∗*^	0.948^*∗*^	0.680^*∗*^	0.303^*∗*^	0.447^*∗*^	1.0^+^	0.752^+^

Second molar	KTP (*n* = 16)	Mean ± SD	7.06 ± 2.23	3.63 ± 2.33	3.38 ± 2.96	2.63 ± 2.47	1.44 ± 1.41	0.81 ± 1.04	0.25 ± 0.44
		Minimum	4	0	0	0	0	0	0
		Maximum	10	8	10	6	4	3	1
	Tablet (*n* = 16)	Mean ± SD	6.13 ± 2.15	3.31 ± 2.57	3.06 ± 2.76	2.31 ± 2.35	1.13 ± 1.25	0.38 ± 0.61	0.06 ± 0.25
		Minimum	4	0	0	0	0	0	0
		Maximum	10	8	7	6	3	2	1
		*P* value	0.237^*∗*^	0.722^*∗*^	0.760^*∗*^	0.717^*∗*^	0.514^*∗*^	0.341^+^	0.381^+^

^*∗*^ *T*-test;^+^Mann–Whitney test.

**Table 3:** Correlation of preoperative pain and postoperative pain separately in each group.

Group		2 hours	4 hours	8 hours	12 hours	24 hours	48 hours
KTP	Correlation coefficient	0.466^*∗∗*^	0.716^*∗∗*^	0.574^*∗∗*^	0.647^*∗∗*^	0.548^*∗∗*^	0.316
	*P* value	0.007	<0.001	0.001	<0.001	0.001	0.078
	Number	32	32	32	32	32	32

Tablet	Correlation coefficient	0.318	0.390^*∗*^	0.189	0.208	0.185	0.069
	*P* value	0.076	0.027	0.300	0.254	0.311	0.709
	Number	32	32	32	32	32	32

^*∗*^Correlation was significant at the 0.05 level of significance. ^*∗∗*^Correlation was significant at the 0.01 level of significance.

## Data Availability

The patients' data used to support the findings of this study are included within the supplementary pdf file, named “data.”
