# Supplementary material for: Analgesic Efficacy of Ketoprofen Transdermal Patch versus Ibuprofen Oral Tablet on Postendodontic Pain in Patients with Irreversible Pulpitis: A Randomized Clinical Trial
Source: Pain Res Manag. 2023 Jun 7;2023:8549655. doi: 10.1155/2023/8549655 (PMC10266914; doi:10.1155/2023/8549655)
Supplement: Supplementary Materials — The pain database of patients can be found in the attached pdf file, named 8549655.f1. [file 8549655.f1.pdf]

code	id	teeth	drug	Anxiety	Pain.before
1	1	6	1	2	4
1	2	6	1	0	7
1	3	6	1	7	7
1	4	6	1	1	4
1	5	6	1	6	10
1	6	6	1	3	8
1	7	6	1	3	7
1	8	6	1	3	5
2	9	6	1	5	3
2	10	6	1	2	5
2	11	6	1	10	10
2	12	6	1	5	4
2	13	6	1	7	8
2	14	6	1	7	7
2	15	6	1	2	8
2	16	6	1	3	9
3	17	6	2	0	4
3	18	6	2	5	3
3	19	6	2	4	8
3	20	6	2	2	8
3	21	6	2	8	7
3	22	6	2	3	6
3	23	6	2	5	5
3	24	6	2	0	5
4	25	6	2	4	6
4	26	6	2	0	4
4	27	6	2	2	8
4	28	6	2	3	4
4	29	6	2	6	4
4	30	6	2	2	5
4	31	6	2	6	8
4	32	6	2	5	4
5	33	7	1	7	9
5	34	7	1	0	4
5	35	7	1	5	10
5	36	7	1	3	8
5	37	7	1	6	7

5	38	7	1	5	6
5	39	7	1	0	5
5	40	7	1	1	9
6	41	7	1	5	5
6	42	7	1	4	10
6	43	7	1	6	7
6	44	7	1	5	5
6	45	7	1	6	5
6	46	7	1	5	9
6	47	7	1	2	4
6	48	7	1	6	10
7	49	7	2	6	5
7	50	7	2	0	4
7	51	7	2	0	4
7	52	7	2	7	7
7	53	7	2	5	10
7	54	7	2	7	7
7	55	7	2	5	4
7	56	7	2	3	5
8	57	7	2	8	8
8	58	7	2	2	4
8	59	7	2	2	5
8	60	7	2	4	4
8	61	7	2	6	6
8	62	7	2	7	9
8	63	7	2	5	10
8	64	7	2	0	6

Pain.2hour	Pain.4hour	Pain.8hour	Pain.12hour	Pain.24hour	Pain.48hour
2	2	1	0	0	0
9	3	2	0	0	0
7	5	3	3	0	0
0	0	0	0	0	0
5	6	5	3	3	1
5	7	8	5	1	0
2	1	0	0	0	0
1	1	0	0	0	0
2	0	0	0	0	0
0	0	0	0	0	0
8	10	5	2	1	0
4	0	0	0	0	0
8	5	5	5	2	0
2	3	2	1	0	0
0	0	0	0	0	0
2	2	1	1	0	0
1	0	0	0	0	0
4	4	2	0	0	0
1	1	0	0	0	0
2	2	2	1	1	0
7	4	1	0	0	0
2	0	0	0	0	0
5	4	1	0	0	0
2	3	2	1	0	0
4	2	0	0	0	0
1	1	1	1	1	0
4	6	2	1	0	0
5	3	3	3	3	1
2	0	0	0	0	0
2	0	0	0	0	0
6	4	1	1	0	0
8	5	5	5	2	2
4	5	5	3	2	0
2	0	0	0	0	0
8	7	3	3	3	1
3	1	0	0	0	0
6	4	4	1	1	1

6	0	0	0	0	0
2	1	0	0	0	0
3	5	5	3	1	0
4	6	6	3	2	1
7	10	5	1	0	0
5	4	2	2	2	0
0	0	0	0	0	0
4	4	6	2	0	0
2	2	1	1	0	0
0	0	0	0	0	0
2	5	5	4	2	1
0	0	0	0	0	0
5	4	5	3	0	0
0	0	0	0	0	0
1	0	0	0	0	0
6	6	2	1	1	0
8	5	5	3	1	0
1	0	0	0	0	0
3	1	0	0	0	0
3	4	6	3	2	0
1	0	0	0	0	0
0	0	0	0	0	0
5	7	5	2	0	0
4	6	3	1	0	0
6	5	2	1	0	0
6	6	5	3	1	1
4	5	4	1	1	0
